# John Percy Ingham Harty

**Published:** 1928

**Authors:** 


### Obituary.

---

### JOHN PERCY INGHAM HARTY, F.R.C.S. Eng.

We deeply regret to record the death of Mr. John Harty at the age of 47, which occurred on March 10th. Mr. Harty was born in Ireland and received his medical education at Queen's College, Cork, and graduated M.B., B.Ch., B.A.O., in the Royal University of Ireland in 1902. After spending several years at Halifax in general practice he decided to specialise in ear, nose and throat work, and studied in Leeds and London. In the remarkably short period of eighteen months he passed both the primary and the final examinations of the Royal College of Surgeons of England, and obtained the diploma of F.R.C.S. in 1912. Then he went back into residence in various hospitals in London and Cardiff, and finally came to Bristol as House Surgeon to the Ear, Nose and Throat Department. Not many men could have re-adjusted themselves to the life of a hospital resident after being ten years in practice in so happy a fashion as Harty. To his fellow-residents he was just one of themselves, and amongst the honorary staff he always seemed to be one of their number also. Harty somehow fitted in wherever he found himself.

Just before the war he was made Registrar of the Ear, Nose and Throat Department at the Bristol Royal Infirmary, and started private consultative work with immediate success.

At the outbreak of the war he was mobilised with the 3rd South Midland Field Ambulance, and went to France with his Division (the 48th) early in 1915. Soon afterwards he was attached to No. 6 General Hospital at Rouen as a specialist, and organised the throat and nose department for the Rouen base. Towards the end of the war he was transferred to the Royal Air Force, and served with it as a specialist until his demobilisation in 1919. On returning to Bristol he resumed his work, and in 1921 was appointed Honorary Surgeon in charge of the Ear, Nose and Throat Department in succession to Dr. P. Watson-Williams, who writes the following appreciation of his lamented colleague :—

"It was with a sense of profound consternation that we, his old colleagues and friends, learnt that John Ingham Harty had succumbed to an operation which it was so earnestly hoped would restore him to good health for many a year. No one was more intimately associated with Harty than I was since he first came to Bristol Royal Infirmary as my House Surgeon in 1913. In 1918 Harty began private practice, and I had the advantage of his collaboration as Clinical Assistant; the following year he was appointed Registrar, but shortly after the outbreak of war his army service claimed him. While serving in France he was appointed surgeon in charge of the special ear, nose and throat clinic of the base hospital at Rouen, and there his skilful work prevented his release till 1920. Meanwhile he had been appointed Assistant Surgeon at the Bristol Royal Infirmary, and in recognition of his outstanding good work this appointment was antedated in view of the fact that he had already been recommended for this appointment to the honorary staff, and it was only due to his army service that the appointment had not been already carried to completion. A matter of form perhaps, nevertheless a sure indication of the warm regard and esteem for an absent colleague, and which I know was a source of happiness to himself. We were all glad when at length he was released to take up afresh his private and hospital practice, apparently in splendid health. How well I recall with what delight and pride he told me of his engagement, 'a romance of the war,' he declared, with that twinkle in the merry blue eyes that looked so straight into one and bespoke that warm heart that made him the beloved colleague and friend to all who knew him. His happy marriage was soon followed by his appointment on my resignation as Surgeon-in-Charge of the Special Department, in which we had worked together in complete harmony for so many years. We all looked on Harty as marked out for a brilliant career, little realising that attacks of illness from exposure during the war had left a legacy at first hardly noticeable as a latent nasal focal sepsis. At first declaring itself by recurrent attacks of rheumatism and lumbago, ere long very painful sciatica developed, for which he underwent operation as well as operative treatment on his nasal sinus infection. He appeared to have recovered good health till further systemic complications caused frequent illness, then appendicectomy by Sir Berkeley Moynihan led to the hope that at length all would be well. Unfortunately, within two years a spreading duodenal ulcer rendered further operation inevitable; nevertheless, with his indomitable courage, he continued the work he loved and adored till within a few days of his last operation and death.

"Harty's long training, first in general surgery and later in his speciality, with a large measure of native talent and wonderful perseverance, would have yielded not only their natural and rightful rewards to himself, but in a larger measure the enrichment of the literature of his speciality than was possible for anyone so repeatedly laid aside by painful illnesses and operations. Nevertheless, despite the difficulties he had to contend with, he took an active share in the work of local medical societies, frequently showing interesting cases that had benefited by his operative treatment and in contributions to the *Bristol Medico-Chirurgical* and other journals."

Another colleague, Dr. R. S. Statham, who was a fellow-resident with Harty at the Bristol Royal Infirmary and in close touch with him at Rouen, has written of him from other aspects :—

"The death of J. P. I. Harty has deprived Bristol of one of its best-known consultants and has caused a gap in the circle of his colleagues and friends which it will be well-nigh impossible to fill. Those who met him for the first time after the war little realised how much it had changed him. A noted Rugby player (he played twice for the South of Ireland and was 'capped' for his medical school all the time he was a student), he played most games well, and was endowed with a magnificent physique. A typical cheery Irishman, with a never-ending fund of good stories and jokes, he was the best of companions, beloved alike by his colleagues and patients. From the date of his first illness in France he never enjoyed good health, and in later years, in spite of numerous operations, he was greatly aged and never free from pain—at times very severe—but always so bravely borne that those who did not know him well seldom had any idea of the distress he was enduring. He 'did great good by stealth.' The shoals of letters received from his poorer patients are an eloquent testimony to the affection he inspired in them."

Mr. Harty married, in 1916, Helen Dorothy, daughter of the late Dr. Clarke, of Kensington, and leaves two young sons, to whom we offer our sincere sympathy.

